# Corneal application of SOCS1/3 peptides for the treatment of eye diseases mediated by inflammation and oxidative stress

**DOI:** 10.3389/fimmu.2024.1416181

**Published:** 2024-07-22

**Authors:** Chulbul M. Ahmed, Howard M. Johnson, Alfred S. Lewin

**Affiliations:** ^1^ Department of Molecular Genetics and Microbiology, University of Florida, Gainesville, FL, United States; ^2^ Department of Microbiology and Cell Science, University of Florida, Gainesville, FL, United States

**Keywords:** uveitis, macular degeneration, diabetic retinopathy, glaucoma, kinase inhibitory region peptides

## Abstract

Several blinding diseases affecting the retina and optic nerve are exacerbated by or caused by dysregulated inflammation and oxidative stress. These diseases include uveitis, age related macular degeneration, diabetic retinopathy and glaucoma. Consequently, despite their divergent symptoms, treatments that reduce oxidative stress and suppress inflammation may be therapeutic. The production of inflammatory cytokines and their activities are regulated by a class of proteins termed Suppressors of Cytokine Signaling (SOCS). SOCS1 and SOCS3 are known to dampen signaling via pathways employing Janus kinases and signal transducer and activator of transcription proteins (JAK/STAT), Toll-like Receptors (TLR), nuclear factor kappa-light-chain-enhancer of activated B cells (NF-κB), mitogen activated kinase (MAPK) and NLR family pyrin domain containing 3 (NLRP3). We have developed cell-penetrating peptides from the kinase inhibitory region of the SOCS1 and SOCS3 (denoted as R9-SOCS1-KIR and R9-SOCS3-KIR) and tested them in retinal pigment epithelium (RPE) cells and in macrophage cell lines. SOCS-KIR peptides exhibited anti-inflammatory, anti-oxidant and anti-angiogenic properties. In cell culture, both Th1 and Th17 cells were suppressed together with the inhibition of other inflammatory markers. We also observed a decrease in oxidants and a simultaneous rise in neuroprotective and anti-oxidant effectors. In addition, treatment prevented the loss of gap junction proteins and the ensuing drop in transepithelial electrical resistance in RPE cells. When tested in mouse models by eye drop instillation, they showed protection against autoimmune uveitis, as a prophylactic as well as a therapeutic. Mice with endotoxin-induced uveitis were protected by eye drop administration as well. R9-SOCS3-KIR was particularly effective against the pathways acting through STAT3, e.g. IL-6 and VEGF-A mediated responses that lead to macular degeneration. Eye drop administration of R9-SOCS3-KIR stimulated production of antioxidant effectors and reduced clinical symptoms in mouse model of oxidative stress that replicates the RPE injury occurring in AMD. Because these peptides suppress multiple pathogenic stimuli and because they can be delivered topically to the cornea, they are attractive candidates for therapeutics for uveitis, macular degeneration, diabetic retinopathy and glaucoma.

## Introduction

Clear vision is a critical necessity for a healthy and productive life. The significance of visual system is underscored by the fact that it takes up about 40% of the sensory input and about 50% of the cerebral cortex for processing the visual information. The eye is prone to developing several degenerative diseases from birth to advanced age through inherited mutations, metabolic changes and environmental factors experienced during life. The concept that the eye is an immune privileged organ appears to be an oversimplification. The eye takes several measures to maintain an immunosuppressive environment (1–3). This includes physical barriers such as tight junctions between vascular endothelial cells and tight junctions between cells of the retinal pigment epithelium (RPE), a single layer of cells surrounding the eye between retina and the choroid that provides the outer blood-retinal barrier (BRB). The RPE plays many crucial roles in maintaining the health of eye, including providing nutrition to photoreceptor cells and clearance of waste material following the visual cycle. Immune regulatory proteins can control activation of retinal microglia and T cell activation (4–6). However, components of the immune system that can recognize and evade initial inflammatory insults include the resident macrophages and dendritic cells (DC) in uvea and cornea, the expression of IgA on cornea, and microglia in retina (2). Receptors that can recognize a danger signal, such as toll-like receptor (TLR) and pattern recognition receptor (PRR) are present in various compartments of the eye, including uvea and cornea. Several inhibitory soluble and membrane-bound effector molecules are in place to suppress inflammation and also to program monocytes and T cells to mediate tolerance, both in the RPE and several compartments of the eye (3, 7). The soluble effectors include transforming growth factor (TGF)-β2, neuropeptide α- melanocyte stimulating hormone (α- MSH), IL-1 receptor antagonist (IL-1RA), thrombospondin (TSP)-1, somatostatin, and prostaglandin (PGE)-2. The membrane-bound effectors include Fas ligand (FasL) and programmed cell death 1 (PD-1). Together, these molecules provide a mechanism to inactivate multiple pathways of inflammation. Microglia within the eye play a critical role in regulating inflammation (8–10). They activate upon any inflammatory stimulus, adopt an amoeboid shape and move to the site of injury for its clearance. The presence of regulatory T cells (Tregs) inside the eye (11, 12) also helps provide an immune suppressive environment by inhibition of systemic immune response and suppressing the antigen presenting cells.

## SOCS1 and SOCS3

**Figure 1:**
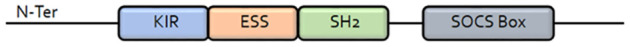
Structural domains of SOCS1 and SOCS3. Variable N terminal regions are followed by kinase inhibitory regions (KIR) that are unique to SOCS1 and SOCS3. These are followed by extended SH2 subdomain (ESS), SH2 domain and a SOCS box toward the C-terminus.

The initiation of an immune response by a cytokine is subject to feedback inhibition by a set of intracellular checkpoint inhibitors called suppressor of cytokines signaling (abbreviated as SOCS). While the immune modulators program cell death 1 cell protein (PD-1) and its ligand and cytokine T lymphocyte antigen 4 (CTLA 4) and its receptor are restricted to cells of the immune system (13), SOCS proteins are present in nearly all cells, including many in different compartments of the eye. SOCS proteins provide a cross talk between the cells of the immune system and non-immune cells (14–17). There are eight members in this group, SOCS1-7 and cytokine inducible SH2 protein (CIS) (14–16). Amongst these, SOCS1 and SOCS3 are homologous and are particularly relevant for the JAK/STAT, TLR, NF-kB, MAPK and NLRP3 signaling (14–16, 18). The structural domains in these proteins are schematized in **Figure 1**. A variable N-terminus is followed by a kinase inhibitory region (KIR), which is unique to SOCS1 and SOCS3, an extended SH2 domain (ESS), the SH2 domain and the SOCS box toward the C-terminus. Structural analysis and deletion analysis reveal that both the KIR and the SH2 domains are required for specific binding of SOCS1 and SOCS3 to their respective target kinases (19, 20).

It is worth noting that the KIR regions of SOCS1 and SOCS3 bind to activation loop of kinases associated with particular JAKs, TLR adapters (21–24), and other regulatory kinases (18) and thus limit the downstream signaling pathways. This binding can occur independently of the rest of the parent SOCS proteins. In addition, in the absence of SOCS box, the KIR-kinase complex is not subject to proteasomal degradation, and the SOCS-KIR peptide is available for multiple rounds of tyrosine kinase binding and inactivation, thus doing “more with less”. Indeed, it was shown for SOCS3 that the full length protein had a half-life of 0.7 hr, while a cell penetrating SOCS3-KIR peptide (devoid of SOCS box) had a half-life of 29 hr (22). The ability of SOCS-KIR 1 and 3 peptides to act on their own has allowed us to develop specific inhibitors to JAK/STAT and TLR2/4, NF-kB, MAPK and NLRP3 pathways by attaching cell penetration moieties (polyarginine, R9 or palmitoyl-lysine) to the N-terminus of KIR regions of SOCS1 and SOCS 3. The resultant peptides are denoted as R9-SOCS1-KIR and R9-SOCS3-KIR, respectively (21, 24–28). The amino acid sequence of SOCS1-KIR is DTHFRTFRSHSDYRRI. Substitution of critical Phe residues with Ala, results in loss of activity and serves as a negative control peptide, which is denoted as R9-SOCS1-KIR2A. A cell penetrating form of SOCS1-KIR peptide has been reported by others to have therapeutic effects in experimental models of atherosclerosis (29), diabetes (30) and diabetic retinopathy (31).

**Figure 2:**
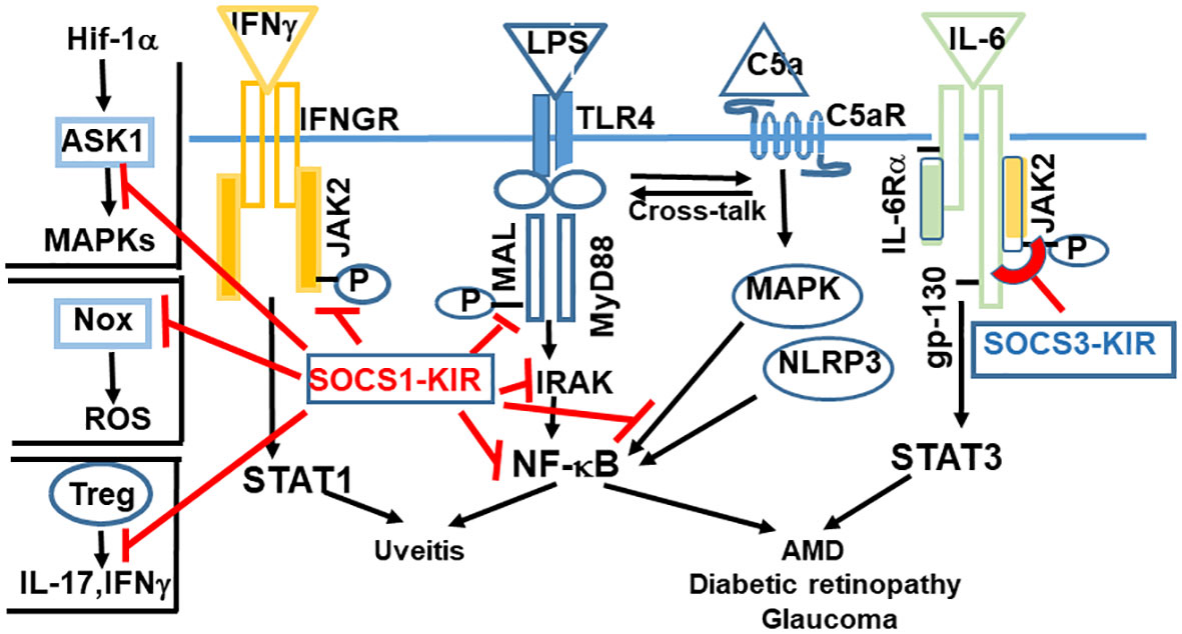
Kinase inhibitory regions (KIR)s of SOCS1 and SOCS3 are negative regulators of inflammation and oxidative stress. SOCS1-KIR binds to JAK2, which leads to suppression of IFNγ signaling. Binding to MAL adaptor protein, IRAK, NF-kB, or MAP kinase results in blocking of signaling initiated by LPS or complement 5a. The functionality of Tregs is enhanced by SOCS1. Binding of SOCS1-KIR to Nox results in anti-oxidant response. SOCS3 is unique in binding to the gp-130 receptor subunit of IL-6 family of cytokines, followed by binding to the corresponding tyrosine kinase. Abbrev: ASK1, Apoptosis Signaling-Regulating Kinase; Hif-1α, Hypoxia inducible factor 1α; Nox, nicotinamide adenine dinucleotide phosphate oxidase; ROS, reactive oxygen species.

The amino acid sequence of R9-SOCS3-KIR is (R9)LRLKTFSSKSEYQLVV. A peptide with a randomized amino acid sequence (R9)KSQYVRLSVLFEKTSL, denoted as R9-control-SOCS3 is used as a negative control peptide. Marasco and co-workers reported the use of a cell penetrable version of SOCS3-KIR for the treatment of cancer (32) in a rodent model. The specific JAKs, receptor subunits, adaptor proteins, or regulatory proteins to which SOCS1-KIR and SOCS3-KIR bind to and the downstream signaling that is blocked is shown in **Figure 2**. **Table 1** shows the specific immune mediators that are affected and the biological outcome. Details of the signaling events and the onset of the corresponding diseases are summarized in **Tables 2**, **3**.

**Table 1:** JAKs or adaptor protein MAL that are affected by SOCS1-KIR or SOCS3-KIR peptide binding are shown in bold letters.

Cytokine or effector	JAKs or Adapter	Biological Effect	SOCS protein involved
IFNγ	JAK1, **JAK2**	Th1 cell induction	SOCS1
IL-12	**JAK2**, **TYK2**	Th1 cell induction	SOCS1
IL-17	**JAK2**	Th17 cell induction	SOCS1
IL-23	**JAK2**, **TYK2**	Th17 cell induction	SOCS1
IL-6	**JAK2**, **TYK2**	Pro-inflammatory	SOCS3
IL-22	**JAK2**, **TYK2**	Endothelial barrier integrity	SOCS3
TNFα	**MAL**	NF-κB signaling	SOCS1/3
LPS	**MAL**	Th1 cell induction	SOCS1/3
VEGF-A	**JAK2**	Neovascularization	SOCS3

The biological response that is affected as a consequence is shown (reviewed in (14–16)).

**Table 2:** Ocular Inflammatory Diseases: Current treatments and their limitations.

Disease	Description	Symptoms	Risk Factors	Treatments	Limitations
**Uveitis**	Inflammation of uveal layer	Redness; pain; blurred vision. Can lead to loss of vision	Ocular infection; underlying autoimmune disease	Control of infection or underlying autoimmune disease; corticosteroid treatment	Prolonged steroid therapy increases the risk of glaucoma
**Wet AMD**	Sprouting blood vessels from the choroidal layer into the central retina (macula); retinal fibrosis	Visual distortion; sudden loss of central vision	Advanced age, genetic risk, smoking, obesity, cardiovascular disease	Anti VEGF-A antibodies or soluble VEGF trap	Does not prevent inflammation. Requires repeated intraocular injections that can lead to infection or inflammation
**Advanced Dry AMD** **(Geographic Atrophy)**	Death of macular photoreceptors and the underlying retinal pigment epithelium	Visual distortion; gradual irreversible loss of central vision	Advanced age, genetic risk, smoking obesity, cardiovascular disease	Anti C3 antibody; anti C5 aptamer	Slows but does not stop degeneration. Requires repeated intraocular injections that can lead to infection or inflammation
**Diabetic Retinopathy**	Damage to retinal blood vessels that may lead to sprouting of leaky blood vessels and scarring (proliferative diabetic retinopathy)	Floaters (dark spots in visual field); blurry or hazy vision; dark or empty areas in the visual field; loss of vision.	Uncontrolled type 1 or type 2 diabetes.	Anti VEGF-A antibodies or soluble VEGF trap. Laser surgery to control hemorrhaging	Does not prevent inflammation. Requires repeated intraocular injections that can lead to infection or inflammation. Laser surgery destroys portions of the retina.
**Glaucoma**	Damage to retinal ganglion cells and the optic nerve	Patchy blind spots in peripheral vision; Eventual loss of central vision	Advanced age; elevated intraocular pressure (IOP)	Medications or surgery (trabeculectomy) to reduce IOP	Medical treatment and surgery eventually fail. Does not prevent inflammation or provide neuroprotection.

**Table 3:** Anti-inflammatory and Anti-oxidant Pathways Affected in Ocular Inflammatory Diseases.

Disease	Inflammatory Pathways Affected	Oxidative Damage and Affected Pathways	SOCS proteins involved
**Uveitis**	JAK2/STAT1, NF-κB, IFNγ, IL-17	Lipid peroxidation, DNA oxidation, Nox, SOD, PI3K, and Hif-1α, Other anti-oxidant factors	SOCS1
**AMD**	JAK2/STAT3, NF-κB, IL-6, Complement	Lipid peroxidation, DNA oxidation, Nox, SOD, PI3K, and Hif-1α, Other anti-oxidant factors	SOCS3
**Diabetic retinopathy**	NF-κB, MAPK/ERK, JNK, Complement, NLRP3	Lipid peroxidation, DNA oxidation, AGE Nox, SOD, PI3K, and Hif-1α, Other anti-oxidant factors	SOCS3
**Glaucoma**	NLRP3, IL-6, Complement	Lipid peroxidation, DNA oxidation, AGE, Nox, SOD, PI3K, and Hif-1α, Other anti-oxidant factors	SOCS3

AGE, advanced glycation end products; Nox, NADPH oxidase; SOD, superoxide dismutase.

SOCS1-KIR binds to the activation loops of JAK2, TYK2, and to the MAL adaptor protein (MyD88 adaptor like) (21, 23, 24, 33). Through their inhibitory effects, the signaling downstream of these tyrosine kinases or the MAL adaptor protein is dampened. For example, binding to JAK2 suppresses the effect of IFNγ (also IL-12), and IL-17 (33, 34). Binding to MAL leads to the suppression of LPS initiated response, downstream of MyD88, including to NF-κB signaling. Similarly, by binding to the MAL protein associated with the adaptor MyD88, SOCS1 inhibits signaling from TLR4, which is responsible for signaling from LPS. SOCS1-KIR also binds to Rac1-GTP and decreases generation of reactive oxygen species (ROS) and the subsequent inflammation from NLRP3 inflammasome (35). Thus, activation from transcription factors STAT1, STAT3 and NF-kB is inhibited, as is the activation of NLRP3 inflammasome.

Inflammasome activation in the retina is characteristic of certain retinal diseases including age related macular degeneration (AMD) and diabetic retinopathy (36, 37). Inflammasomes (NLRP1 and NLRP3) are cytosolic multiprotein complexes that detect danger signals in the innate immune system. Amongst these, NLRP3 has been implicated in the pathologies associated with autoimmune disorders (38) and retinal degenerative disorders (39). NLRP3 activation leads to the secretion of IL-1β and IL-18. IL-1β acts through IRAK4, which is inhibited by SOCS1-KIR. The signaling from IL-18 is mediated by MyD88, which is also inhibited by SOCS1-KIR. Thus, SOCS1-KIR dampens the damage caused by continuous NLRP3 activation. The net effect of binding to these kinases is a reduced production of pro-inflammatory effectors, which is beneficial in combating an inflammatory response.

Regulatory T cells (Tregs) play an important role in maintaining immune homeostasis. SOCS1 has been shown to be required for maintaining the expression of Foxp3 in Tregs (40). Tregs do not ordinarily secrete inflammatory cytokines, but in the absence of SOCS1, Tregs secrete IFNγ and IL-17 by hyperactivation of STAT1 and STAT3 (40). The enhancement of Treg function is likely to have a neuroprotective role in retinal diseases. In addition, SOCS1 also inhibits expression of crucial immune molecules on cell surface, including MHC class I, class II and CD40, promoting a general dampening of T cell activation and limiting migration of inflammatory cells. SOCS1 has also been shown to bind to MAP kinase, Apoptosis Signaling-Regulating Kinase (ASK) and limit signaling from c-Jun N-terminal kinase (JNK) pathway (p38 and MAP kinase) (41) (**Figure 2**; **Table 3**). SOCS1 binds to the NADPH oxidase (Nox) subunits and can induce anti-oxidant enzymes (42).

Macrophages can change their phenotype based on the cellular milieu (18). The classically activated M1 macrophages produce high levels of inflammatory cytokines, whereas M2 macrophages can have neuroprotective and healing roles. Cytoplasmic release of mitochondrial DNA produced from chronic oxidative stress and inflammation has been shown to have an inflammatory effect in AMD by enhancing the production of IL-6, IL-8, and NLRP3 (43).

It has been suggested that SOCS1 functions mostly to inhibit signaling by interferons (both type I and type II), mediated by STAT1, while SOCS3 is involved predominantly in inhibition of signaling by IL-6 family of cytokines, acting through STAT3 (**Figure 2**, **Tables 1**, **3**) (15, 44). Specific kinase domains of JAKs or their adaptors are the target sites for SOCS1. In contrast, the gp130 subunit of IL-6 family of receptors is initially the docking site for SOCS3, from which it is transferred to kinase domains of specific JAKs (**Figure 2**). This distinction is not strict, since there is still weaker inhibition of STAT3 signaling by SOCS1 and suppression of STAT1 by SOCS3. Simvastatin treatment of patients with multiple sclerosis (MS) leads to elevation in SOCS3 levels. This elevation correlates with reduced STAT3 phosphorylation and diminished levels of IL-6 and IL-17 (45, 46), providing further evidence of their signaling through STAT3. In addition, SOCS3 was also shown to suppress signaling from IL-1β (47, 48). Within the eye, SOCS3 expression has been observed in several compartments including the retina (49). In SOCS3-deficient retinas, degradation of rhodopsin leading to loss of photoreceptor cells and ensuing impaired visual function were noted, due to an overactive STAT3 (50, 51).

Cell penetrating peptides (CPPs) provide a method to deliver therapeutic peptides to tissues as an alternative to delivery by gene therapy vectors (52, 53). Microbial production of recombinant proteins or chemical synthesis permit production of peptides in quantities sufficient for human therapy. Naturally occurring cell penetration peptides, such as the HIV tat_49–57_ as well as polyarginines permit transport of coupled proteins across the plasma membranes of cells (54). Conjugation with palmitoyl-lysine is an alternative approach to promote transport through lipid bilayers. For example, Jager et al. demonstrated that a cell penetrating version of SOCS1-KIR traversed the blood-brain barrier and protected mice from experimental allergic encephalomyelitis (EAE), a mouse model of multiple sclerosis (MS) (21).

## Complement

Activation of the alternative complement pathway contributes to the inflammatory response in AMD, diabetic retinopathy and glaucoma (55–58). Complement proteins, in addition to oxidized lipid and carbohydrate waste, are found in drusen, the sub-RPE deposits that are pathognomonic of dry AMD. The cleaved components of the complement C5 and C3, C5a and C3 proteins are known as anaphylatoxins. These promote inflammation, attract active mast cells, increase cell permeability, and induce cell adhesion molecules that further recruits neutrophils and monocytes (57, 59). Formation of the membrane attack complex (MAC) with complement activation can lead to the death of RPE cells, resulting in increased permeability and further damage to the outer retina (60). In combination with MAC, C3a and C5a can also induce inflammasome signaling (61–63). In addition, C3a and C5a can promote Th17 cell differentiation by inducing IL-17, which plays a vital role in the pathology of both dry and wet AMD (64, 65). Complement activation has also been shown to induce VEGF-A, which facilitates the choroidal neovascularization (66). The endogenous complement factor H (CFH) naturally inhibits the complement pathway. Mutations in CFH that result in loss of its ability to put brakes on complement pathway constitutes 50% of the genetic risk of AMD (67). C3a and C5a act through their cognate G protein coupled seven transmembrane receptors, C3aR and C5aR, respectively, activating the MAP kinase followed by signaling through NF-kB. (**Figure 2**). A cross-talk between complement and TLR signaling has been reported (68), which activates NF-kB pathway. Oxidative stress enhances the effect of complement activation (69). While complement is a part of the innate arm of the immune system, in AMD, transport of the IL-17 producing γδT cells was reported (56, 64), suggesting the involvement of both innate and adaptive arms of the immune system. The United States Food and Drug Administration has approved two complement inhibitors, Syvore™, which inhibits C3, and Izervay™, a complement C5 inhibitor, for the treatment of geographic atrophy, the advanced form of dry AMD (see below). In Europe, the EMA has approved Izervay™ but not Syvore™.

Overexpression of SOCS-3 inhibited complement activation in renal tubule cells treated with calcineurin inhibitor (70). We have shown that treatment with R9-SOCS3-KIR peptide resulted in protection against the damaging effects of C5a by its ability to suppress NF-κB pathway, which is a major producer of inflammatory cytokines, as well as its ability to suppress VEGF-A secretion (28). R9-SOCS3-KIR was also shown to down regulate the expression of the inflammatory cytokines IL-1β, IL-6, IL-17A and the chemokine MCP-1. It also prevents the loss of tight junction proteins in RPE cells and the subsequent decrease in transepithelial electrical resistance across the RPE monolayer (28). Loss of tight junction proteins will disrupt the blood-retinal barrier and allow inflammatory cells to breach this barrier. Thus, R9-SOCS3-KIR can protect against the toxicity of C5a at multiple steps.

There are four major eye diseases that arise from or are exacerbated by common effectors including inflammation, oxidative stress and angiogenesis: uveitis, age related macular degeneration (AMD), diabetic retinopathy (DR) and glaucoma. For the treatment of these diseases, we propose the topical delivery of cell penetrating peptides R9-SOCS1-KIR and R9-SOCS3-KIR.

## Uveitis

Uveitis is the fourth leading cause of blindness worldwide (71). It can affect individuals of all ages and is thus a major socio-economic burden. Based on the etiology, there are two types uveitis known: infectious uveitis and autoimmune uveitis. Both these forms of uveitis cause inflammation of the uveal layer, which consists of the choroidal layer surrounding the eye, the ciliary body and the iris. Although uveitis begins with inflammation of uvea, it can spread to other compartments of the eye. Based on the site that is affected, uveitis is classified as anterior uveitis when the anterior chamber is involved, intermediate uveitis where the vitreous cavity is affected, or posterior uveitis when the choroid and retina are involved. When all of these compartments are affected, that is defined as panuveitis (72, 73). Anterior uveitis is characterized by redness, pain and photophobia. Upon examination by slit lamp, white blood cells can be seen in the aqueous humor. This form of the disease is typically short lived and responds to anti-inflammatory therapy. Symptoms of posterior uveitis include the presence of exudate and inflammatory cells, retinal edema, or retinal detachment, all of which lead to varying degrees of blindness. Histological analysis shows disorganized retinal architecture, damage to ganglion and photoreceptor cell layers, retinal folds, sub-retinal exudate, vasculitis, damage to retinal pigment epithelium, and choroiditis (74), thus affecting every compartment of the eye. Left untreated, uveitis can lead to complications such as cystoid macular edema, cataract, secondary glaucoma, vitreous opacities, and retinal scars (75).

Uveitis is often initiated by ocular infection, though it is also commonly associated with underlying autoimmune disease. In a mouse model of uveitis, lipopolysaccharide (LPS), derived from Gram negative bacteria is injected intravitreally, leading to an acute response, restricted mostly to the anterior chamber, and self-resolving symptoms (76). This model shares many features of the damage caused by infection with many pathogens. The LPS model gained greater traction after it was revealed that independent of the exogenous infection, the microbiota from the intestine or oral cavity can also trigger or exacerbate uveitis (77–79). Analysis of intraocular expression of cytokines in uveitis patients has revealed the presence of increased levels of inflammatory cytokines (IFNγ and IL-17) and decreased regulatory T cells (Tregs) (78). Increased expression of IFNγ (Th1), which induces chemokines, results in breakdown of the blood-retinal barrier. Th17 cells, which are induced by IL-23, are another subset of inflammatory cells that are relevant to human uveitis. It is well established that either the Th1 or the Th17 response is capable of causing uveitis (78). Neutralization of Th1 response leads to an elevated Th17 response, and a deficiency of Th17 response leads to an elevated Th1 response (80). This phenomenon has also been observed in clinical experience, where Th17 targeted therapy for Behcet’s uveitis (81), or Crohn’s disease (82) has shown limited success. Thus, a simultaneous blockage of both Th1 and Th17 responses is crucial to the treatment of autoimmunity. A decrease of immunosuppressive regulatory T cells (Tregs) has been observed in uveitis (83), and approaches that enhance Tregs may have therapeutic value.

The current treatments for non-infectious uveitis include corticosteroids, general immunosuppressants, or specific antibodies. Although initial inflammation is suppressed, in cases of recurrent uveitis continued treatment with corticosteroids is associated with development of cataracts, glaucoma, retinopathy, and activation of herpes simplex virus (84). Immunosuppressive agents, such as Cyclosporine A (CsA), a T cell targeting drug that blocks IL-2 signaling has been used for ocular inflammation. FK-506 (tacrolimus) and rapamycin, which also target IL-2 signaling have also been employed. This line of treatment is discouraged, because of the involvement of IL-2 in maintenance of Tregs (85, 86) and decreased levels of Tregs are detrimental. Furthermore, these treatments are also not advised for a prolonged treatment. Clinical use of monoclonal antibodies that bind the tyrosine kinase JAK has therapeutic benefit for the treatment of autoimmune uveitis (87), and refractory uveitis and scleritis (88). In addition, small molecule inhibitors of JAK (89), and TYK2 (90) have been used to treat psoriasis.

We propose the topical use of R9-SOCS1-KIR (R9 indicates nine arginine residues) for the treatment of both infectious and autoimmune uveitis, based on our findings in cell culture and in mouse models (26, 27). The advantages that this treatment offers are several fold: R9-SOCS1-KIR can suppress both Th1 and Th17 responses. It can enhance M2 type of macrophage that have neuroprotective properties, while simultaneously decreasing M1 macrophage polarization (inflammatory) (18, 91). In the absence of SOCS1, Tregs can go on to formTh1 or Th17 cells. There is cross talk between SOCS1 and Tregs such that the protective Tregs are maintained in the presence of SOCS1 (25, 92, 93). We note that Rudensky and colleagues reported that during development, induction of Foxp3 stimulates expression of miR155, which promotes fitness and proliferation of Treg cells by causing SOCS1 down-regulation. However, several groups have found a therapeutic role for SOCS1 in maintaining the desired levels of Tregs (40, 92, 93).

In cell culture studies, we have shown that R9-SOCS1-KIR can: (a) downregulate the expression of inflammatory cytokines, IL-1β and IL-6 and chemokine CCL-2; (b) suppress the transcription initiated by STAT1, MAP kinase p38 and NF-κB; (c) inhibit the secretion of inflammatory cytokines from the RPE cell line ARPE-19 and monocytic cell line THP-1; (d) suppress of secretion of nitric oxide and IL-1β, which would reduce the expression of cyclooxygenase-2 and prostaglandin; (e) protect retinal pigment epithelial cells from TNFα and IL-17A induced loss of tight junction proteins and the ensuing loss of transepithelial electrical resistance (26, 27). In addition, in a mouse model of autoimmune uveitis using B10.RIII mice that were immunized with a peptide from interphotorecptor retinal binding protein (IRBP 161-180), we have shown protection of the retina as seen by preservation of the structural as well as functional features of the eye monitored by fundoscopy, optical coherence tomography, and by electroretinography (ERG) (26). Protection occurred at both the initiation and the effector phases of the disease symptoms. Using a peptide with an amino-terminal palmitoyl-lysine adduct for cell permeation, Egwuagu and colleagues also showed that topical application of SOCS1-KIR could prevent pathology in rodent models of autoimmune uveitis, if delivered from the time of immunization (94, 95).

In cell culture studies, R9-SOSC1-KIR inactivated the NF-kB and the MAP kinase p-p-38 induction by LPS. It also downregulated the synthesis of IL-1β, IL-6, MCP-1, TNFα, iNOS and COX-2. The secretion of IL-1β and NO in a macrophage cell line were also suppressed in the presence of R9-SOCS1-KIR. R9-SOCS1-KIR also protected against the loss of tight junction proteins and the decrease in transepithelial electrical resistance in differentiated retinal pigment epithelial cells. For the infectious uveitis study the endotoxin LPS was used. Uveitis was induced in mice by intravitreal injection of LPS. Eye drop instillation of R9-SOCS1-KIR protected mice as evidenced by fundoscopy, optical coherence tomography (OCT) and histology (27).

Individuals with HLA-B27 haplotype have been reported to have a greater likelihood of developing uveitis (96). Prophylactic treatment with R9-SOCS1-KIR in these individuals is likely to slow the progression of disease. It is worth emphasizing that the benefits obtained in both the autoimmune and endotoxin induced mice were with the R9-SOCS1-KIR delivered by eye drop instillation. Such eye drops will limit any systemic side effects, while allowing the patient the choice of self-administering.

## Age related macular degeneration

In developed countries, age related macular degeneration (AMD) is the leading cause of loss of central vision in the elderly with about 50 million people afflicted worldwide. Its incidence is expected to rise in the coming years because of longer lifespans (97, 98). The macula is a cone photoreceptor rich area in the central retina that contains the fovea and mediates sharp vision. Inflammation and oxidative stress are the major drivers of AMD (99). A hallmark of AMD is the presence of deposits called drusen under the retinal pigment epithelium (100). Analysis of drusen reveals the presence of byproducts of inflammation, complement activation products and oxidized lipid and carbohydrate waste products (55). As the injury to the retinal pigment epithelium continues, blood-retinal barrier is breached, resulting in the entry of cytokines, chemokines and activation of microglia and choroidal dendritic cells. Advanced AMD occurs in two forms, dry (atrophic, or non-neovascular) and wet (neovascular). Wet AMD occurs in about 10% of the cases of AMD, and is characterized by the growth of new and defective blood vessels, often causing hemorrhage with permanent loss of central vision and scar formation over the site of destroyed macula. Vascular endothelial growth factor (VEGF) signaling contributes to the neovascular AMD. C3a and C5a were reported to increase VEGF-A expression leading to increased angiogenesis (66). An overactive complement system is a major contributor of AMD (55–57, 101, 102). Anaphylatoxins C5a and C3a, the products of alternate complement pathway have been reported in drusen and in sera of patients with AMD (56, 60, 66, 103). C5a was reported to promote expression of IL-17 and IL-22. Increased levels of IL-17 and IL-22 were also reported in AMD patients (64). We have already shown the suppression of IL-17 by R9-SOCS3-KIR (28). The signaling by IL-22 proceeds through JAK2/STAT3, which is likely to be suppressed by R9-SOCS3-KIR, making R9-SOCS3-KIR a potential candidate for the treatment of AMD. As noted above, the FDA has recently approved the intravitreal administration of C3a inhibitor, pegcetacoplan (104), and C5a inhibitor, avacincaptad pegol (105). For the wet AMD, frequent intravitreal injections of decoy receptors or VEGF antibodies (106, 107) are employed. Blocking VEGF does not address the ongoing inflammatory damage, and inhibiting the function of VEGF-A entirely may affect the role of basal levels of endogenous VEGF-A (108, 109).

We have reported that the use of R9-SOCS3-KIR peptide can suppress the different pathways that lead to both dry and wet AMD. In cell culture, R9-SOCS3-KIR suppresses inflammatory, oxidative and angiogenic responses (28). As mentioned, above, SOCS3 preferably acts through suppressing the signaling from STAT3. R9-SOCS3-KIR prevents many of the pathways that are mediated by STAT3, which include inhibition of IL-17 and VEGF-A. The following effects of R9-SOCS3-KIR were noted in cells derived from retinal pigment epithelium as well as the mouse macrophage cell line J-774A.1, which were used as surrogate for microglia in the eye. In the section under complement above, we have discussed the ability of R9-SOCS3-KIR to protect against an activated complement. The many damaging properties of anaphylotoxic C5a in cells including induction of NF-κB promoter, nuclear translocation of p65 subunit of NF-kB promoter, induction of inflammatory mediators and secretion of VEGF-A, loss of gap junction proteins and ensuing decrease in transepithelial electrical resistance were all dampened in the simultaneous presence of R9-SOCS3-KIR. In addition, R9-SOCS3-KIR prevented the inflammatory damage caused by LPS or TNFα. R9-SOCS3-KIR also prevented oxidative damage caused by paraquat, with simultaneous induction of anti-oxidant enzymes. In a mouse model of oxidative damage initiated by sodium iodate administration, eye drop instillation of R9-SOCS3-KIR led to protection of the retinal structure and induced the production of anti-oxidant enzymes. Thus, establishing a dose-response relationship and testing the eye drop delivery of R9-SOCS3-KIR in a larger animal model seems warranted.

## Diabetic retinopathy

Diabetic retinopathy (DR) is a complication of ongoing diabetes, and is a leading cause of vision loss in working-age adults worldwide (110, 111). Diabetic retinopathy arises from the sprouting of small blood vessels in the retina. Abnormally high blood sugar coupled with high energy demand of the retina make the situation worse. The high glucose environment is considered akin to hypoxia (112). An intraocular oxygen gradient has been reported in proliferative diabetic retinopathy (113). Hypoxia is sensed by hypoxia-induced transcription factor 1a (Hif-1α) and NF-kB that regulate the synthesis and secretion of inflammatory mediators and growth factors, including VEGF (114–117). Prolonged hyperglycemia leads to glycation of proteins, lipids and nucleic acids, with the resulting products that are known as advanced glycation end products (AGE). AGE are recognized by their cognitive membrane bound receptors known as RAGE. Activation of RAGE leads to enhanced oxidative stress and inflammation because of the activation of NF-κB, MAPK/ERK and JNK pathways (reviewed in (118, 119)). About a third of the diabetics go on to develop diabetic retinopathy. Approximately one third of these develop a severe form of the disease, that includes vascular hemorrhage and neovascularization (120). As of 2023, 100 million patients worldwide were reported to be suffering from the complications of DR and the number is continuing to rise because of the current epidemic of obesity. Individual lifetime risk of diabetic retinopathy is 50-60% in type 2 diabetes (T2D) and 90% in those with type 1 diabetes (T1D). It is becoming increasingly clear that chronic inflammation, oxidative stress, neovascularization, and metabolic changes secondary to hyperglycemia are the major causes of the damage to neuronal and vascular components of the eye. Induction of pro-inflammatory cytokines, chemokines, and adhesion molecules leads to microglial activation, breach of blood-retinal barrier, and the ensuing infiltration of macrophages and other inflammatory cells, followed by neovascularization (121). A combination of these damages leads to changes in contrast sensitivity, delayed dark adaptation, abnormal visual fields, and decrease in visual acuity and electroretinographic amplitudes. Damage to retinal neurons as well as retinal capillaries has been observed. Progressive DR may result in fluid accumulation in the macula, causing swelling known as diabetic macular edema (DME) (37), non-proliferative diabetic retinopathy (NPDR), proliferative diabetic retinopathy (PDR), or a combination of these. PDR is associated with pathological neoangiogenesis, vitreous hemorrhage and retinal detachment.

Increased production of reactive oxygen species (ROS) has been reported in diabetic retinas. This leads to increased activation to NLRP3 and NF-kB pathways, which results in production of inflammatory cytokines (TNFα, IL-1β and IL-6), chemokines (MCP-1, IL-8), adhesion molecules (ICAM-1, VCAM-1) and nitric oxide (NO). Hyperglycemia induced damage to mitochondria results in greater production of ROS. Oxidative damage to mitochondrial DNA leads to impaired transcription of mitochondrial DNA, which results in altered production of electron transport chain proteins, leading to an exacerbation of ROS production (122). Inhibition of superoxides also inhibits glucose-induced production of pro-apoptotic cytochrome c and Bax, which underscores the contribution of mitochondrial oxidative stress to pathologies seen in DR. VEGF is a principal mediator of neovascularization and permeability changes in diabetes. An activated complement cascade has been reported in DR (123), which further worsens the ongoing inflammation.

There is evidence that molecules produced as a result of prolonged hyperglycemia can activate microglia (124). Current data suggest that dysregulated microglial responses are linked to their deleterious effects in several neurological diseases associated with chronic inflammation (125). As inflammatory cytokines and hyperglycemia disseminate through the diabetic retina, microglia can change to an activated state, increase in number, translocate through the retina, and themselves become the producers of inflammatory and apoptotic molecules. Increased content and activation of macrophages has been observed in obesity and diabetes (126, 127), which suggests that suppression of macrophage activation may be a useful strategy to limit the harm done by activated macrophages during chronic inflammation. Toll-like receptors 2 and 4 expression is elevated in macrophages and metabolic tissues during obesity and diabetes (128). TLR4 is also activated because of lipopolysaccharide (LPS) released from microbiota in diabetes (129).

The current treatments for DR are limited and mostly address the late stage of the disease. Use of laser-induced photocoagulation (130) offers some benefits, but it can destroy parts of the retina. Intravitreal injections of anti-vascular endothelial growth factor therapies, or corticosteroids (131), can slow down neovascularization and retinal edema. These treatments require frequent visits to the clinic and are effective in only a subset of patients, suggesting the involvement of alternate pathways in these pathologies. Use of corticosteroids is limited by the observation that it can lead to cataract formation and increased intraocular pressure (132).

As mentioned above, we have shown that R9-SOCS3-KIR exhibits multiple activities that can suppress: (a) inflammation arising from activated complement, LPS, and TNFα; (b) oxidative stress arising from paraquat; and (c) suppress the STAT3 mediated induction of VEGF (28). More importantly, corneal eye drop instillation of R9-SOCS3-KIR protected eyes in a mouse model of severe oxidative stress, as evidenced by histological and other clinical parameters. Suppression of VEGF in the presence of SOCS3 has independently been shown by others (49). It is worth noting that hypoxia resulting in DR from hyperglycemia decreases SOCS3 and increases STAT3 activation followed by up-regulation of VEGF expression (133). These authors further identified a STAT3 responsive promoter upstream of the VEGF gene. Also, a deficiency in SOCS3 has been reported to promote M1 macrophage polarization (134), which will exacerbate the ongoing inflammation. Thus, R9-SOCS3-KIR peptide will serve a triple role of providing the activity of SOCS3 that is lacking, preventing M1 macrophage polarization and providing fresh source of this therapeutic peptide. The use of cell permeable SOCS1-KIR by eye drop delivery and protection against the symptoms has been reported in db/db mice a model of Type 2 diabetes (31). Additionally, topical delivery of peptides derived from PEDF was shown to protect Ins2(Akita) mice from diabetic retinopathy related injuries (135). These examples further suggest that corneal application is a viable option for therapeutic delivery of these peptides. Considering the other benefits we have noted, we propose that R9-SOCS3-KIR is a potential candidate for developing as a therapeutic for diabetic retinopathy.

## Glaucoma

Worldwide, glaucoma (136) is the leading cause of irreversible blindness. Because primary open angle glaucoma (POAG) is a disease of aging, it is estimated that the number of people with glaucoma worldwide will increase to 112 million by the year 2040, resulting from increasing life-span (137, 138). Although increased intraocular pressure (IOP) is considered the major risk factor for developing glaucoma, the disease may occur without an increased IOP. Current therapy for POAG employs eye drops to reduce IOP that must be delivered daily. Compliance is an issue with these treatments, and eventually most of the IOP lowering drugs fail. Degeneration of retinal ganglion cells, loss of their axons and the progressive damage of the optic nerve head accompanied by visual field impairments are the main clinical features of glaucoma (139, 140). Evidence from animal models and patient studies have suggested that inflammation (58, 141), chronic oxidative stress (142–144), mitochondrial dysfunction (144) and hypoxia (139, 145) are the contributing factors leading to glaucoma.

As noted above, chronic oxidative stress can give rise to oxidized proteins, lipids and lipoproteins and metabolic products known as advanced glycation end products (AGE) (146). Increased IOP is believed to decrease the oxygen supply to the retinal ganglion cells, leading to an hypoxic state (145). Hif-1α, which induces nicotinamide adenine dinucleotide phosphate oxidase 2 (NOX-2) and inducible nitric oxide synthetase (iNOS) resulting in increased reactive oxygen species (ROS) production. ROS can, in turn, increase the expression of Hif-1α, creating a feedback loop that increases inflammation and apoptosis (147). In glaucoma, large, active (amoeboid) microglia localize to the parpaillary region of the optic nerve head (148). Activation of microglia leads to secretion of TNFα (149) and activation of the transcription factor NF-κB, which stimulates the synthesis of several inflammatory factors, including IL-1β and IL-6 (150), that can damage the retina. Induction of TLRs (151, 152) and NLRP3 have also been reported in glaucoma (153, 154). Enhancement of several complement factors including C1q, C3, C5 and membrane attack complexes have been observed in glaucoma patients and in animal models of glaucoma (155–157). Intravitreal treatment with antibody to C5 was shown to reduce retinal ganglion cell loss and prevent the degenerative symptoms noted in glaucoma (158). A trend toward downregulation of complement factor H (CFH) was reported in patients with glaucoma (159). Reducing CFH would indicate a greater susceptibility of retinal ganglion cells to complement mediated lysis in glaucoma.

As mentioned above, R9-SOCS1-KIR and R9-SOCS3-KIR have shown protection against damage from inflammation and oxidative stress in cells derived from RPE as well as in macrophages, and protection in the eye in mice with an increase in anti-oxidant factors and neurotrophic factors following topical application (27, 28). Since inflammation and oxidative stress can be alleviated by R9-SOCS1-KIR or R9-SOCS3-KIR, it is plausible that these cell-penetrating peptides may have therapeutic benefit in the treatment of glaucoma. In support of this conjecture, anti-inflammatory and anti-oxidant nutrients that have shown therapeutic benefits in animal models of glaucoma (154, 160). We have already reported the ability of R9-SOCS3-KIR to suppress the cumulative damage from inflammation through these pathways, thus suggesting the use of R9-SOCS3-KIR as a potential therapeutic candidate for glaucoma.

## Conclusions

The four different degenerative eye diseases we have described have common origins in inflammation, oxidative stress and neovascularization. The cell penetrable peptides R9-SOCS1-KIR and R9-SOCS3-KIR we have developed and tested can counter these multiple targets and rescue the ongoing symptoms in animal models of eye disease. These peptides can be modified to have a longer half-life. These peptides need to be tested in animal models with a defined macula, and their pharmacokinetics be carried out for further development. The current anti-VEGF treatments for AMD and DR involve regular visits to the clinic for intravitreal injections with the likelihood of infections or other side effects. In contrast, the peptides we are proposing can be self-administered by the patient, and, since they do not have access to any other organs, they are unlikely to cause systemic side effects.

## Author contributions

CA: Investigation, Writing – review & editing, Conceptualization. HJ: Conceptualization, Funding acquisition, Writing – review & editing. AL: Conceptualization, Funding acquisition, Project administration, Resources, Supervision, Writing – review & editing.
